# Supplementary material for: Integrated multi-omic analysis of pediatric metastatic osteosarcoma reveals endothelial cell plasticity and lineage infidelity
Source: bioRxiv. 2026 Aug 12:2026.08.11.744222. Preprint. [Version 1] doi: 10.64898/2026.08.11.744222 (PMC13640707; doi:10.64898/2026.08.11.744222)
Supplement: Supplementary file 2 [file media-2.pdf]

Sample_Name	Disease Status	Tissue	Age	Treatment Status
CCDI0054n59_1535	Metastatic	Para-aortic Lymph Node	12	post-treatment
CCDI0046n64_1600	Metastatic	Liver	17	post-treatment
CCDI0094_7977	Metastatic	Lung	16	post-treatment
CCDI0095_7978	Metastatic	Lung	16	post-treatment
CCDI0090_2318	Metastatic	Lung	11	post-treatment
CCDI0048_5864	Metastatic	Lung	14	post-treatment
CCDI0049_5899	Metastatic	Lung	14	post-treatment
CCDI0050_5928	Metastatic	Chest Wall	14	post-treatment
CCDI0051_6547	Metastatic	Lung	17	post-treatment
CCDI0091_3666	Metastatic	Lung	16	post-treatment
CCDI0092_3911	Metastatic	Lung	16	post-treatment
CCDI0093_6546	Metastatic	Lung	17	post-treatment
CCDI0099_5913	Metastatic	Chest Wall	14	post-treatment
CCDI0100_12416	Metastatic	Lung	21	post-treatment
CCDI0096_8952	Metastatic	Lung	10	post-treatment
CCDI0098_8951	Metastatic	Lung	10	post-treatment
CCDI0097_8978	Metastatic	Lung	21	post-treatment
CCDI0052_11487	Metastatic	Superior Sulcus	No data	No data
CCDI0053_445	Metastatic	Lung	15	No data
CCDI0055_1804	Metastatic	Mediastinum	12	post-treatment
CCDI0057_16178	Metastatic	Lung	11	No data
CCDI0058_16183	Metastatic	Lung	11	No data
CCDI0101_16178b	Metastatic	Lung	11	No data
CCDI0102_16183b	Metastatic	Lung	11	No data
