# Supplementary material for: Integrated multi-omic analysis of pediatric metastatic osteosarcoma reveals endothelial cell plasticity and lineage infidelity
Source: bioRxiv. 2026 Aug 12:2026.08.11.744222. Preprint. [Version 1] doi: 10.64898/2026.08.11.744222 (PMC13640707; doi:10.64898/2026.08.11.744222)
Supplement: Supplementary file 3 [file media-3.pdf]

Reagent	RRID	Vendor
20X Nuclei buffer	---	10X Genomics
Goat Anti-GFP Polyclonal Antibody	RRID:AB_305643	Abcam
Alvetex 3D scaffolds Amsbio AMS.AVP004-96	---	Ambbio
TdTomato (E3G5L) Rabbit Monoclonal Antibody	RRID:AB_3662938	Cell Signaling Technologies
5M NaCl	---	Corning
3 mM cell insert for 6-well plate	---	Corning
Gelatin coated 6-well plate	---	Corning
Matrigel, Phenol red-free, growth factor reduced	---	Corning
HyClone Bovine Growth Serum (BGS)	---	Cytiva
Bovie Serum Albumin (BSA)	---	GoldBio
1M $MgCl_2$	---	Invitrogen
5% Digitonin	---	Invitrogen
PBS	---	Invitrogen
IMDM, GlutaMAX Supplement	---	Invitrogen
Penicillin-Streptomycin (5,000U/mL)	---	Invitrogen
GelTrex LDEV free reduced growth factor (15 mg/ml)	---	Invitrogen
Opti-MEM Reduced Serum Medium	---	Invitrogen
Hank's Balanced Salt Solution, no calcium, no magnesium	---	Invitrogen
DMEM, high glucose, GlutaMAX Supplement	---	Invitrogen
AO/PI	---	Logos Biosystems
OGM Osteoblast Growth Medium BulletKit	---	Lonza
EGM-2 MV Microvascular Endothelial Cell Growth Medium-2 BulletKit	---	Lonza
Human lung microvascular endothelial cells (HMVEC-L)	---	Lonza
1M DTT	---	Millipore Sigma
RNase inhibitor	---	Millipore Sigma
4-hydroxytamoxifen	---	Millipore Sigma
gentleMACS C tube	RRID:SCR_020270	Miltenyi
gentleMACS Octo dissociator	RRID:SCR_020271	Miltenyi
MACS SmartStrainers (70 µm) (Miltenyi #130-098-462)	---	Miltenyi
MACS SmartStrainers (30 µm) (Miltenyi #130-098-458)	---	Miltenyi
NEBNext Ultra II Directional RNA Library Prep Kit for Illumina	---	New England Biolabs
NEBNext Poly(A) mRNA Magnetic Isolation Module	---	New England Biolabs
NEBNext® Multiplex Oligos for Illumina® (96 Unique Dual Index Primer Pairs)	---	New England Biolabs
Fetal Bovine Serum (FBS), Heat Inactivated	---	Omega Scientific
1M Tris-HCl pH 7.5	---	Quality Biological
Nuclease-free H_2O	---	Quality Biological
Recombinant Human BMP-4 Protein	---	R&D Systems
Corn Oil	---	SelleckChem
Formical-4 Decalcifier	---	StatLab
OS186 Human Osteosarcoma Cell Line	RRID:CVCL-C8FT	Alejandro Sweet-Cordero (UCSF)
OS742 Human Osteosarcoma Cell Line	RRID:CVCL_C8FY	Alejandro Sweet-Cordero (UCSF)
OS052 Human Osteosarcoma Cell Line	RRID:CVCL_C8FR	Alejandro Sweet-Cordero (UCSF)
OS384 Human Osteosarcoma Cell Line	RRID:CVCL_C8FU	Alejandro Sweet-Cordero (UCSF)
BLOXALL Blocking Solution	RRID:AB_2336257	Vector Laboratories
Normal Horse Serum Blocking Solution	RRID:AB_2336617	Vector Laboratories
Antigen Unmasking Solution, Citric Acid Based	RRID:AB_2336226	Vector Laboratories
ImmPRESS-HRP Anti-Rabbit IgG Polymer Detection Kit	RRID:AB_2336529	Vector Laboratories
ImmpACT 3,3'-diaminobenzidine	RRID:AB_2336520	Vector Laboratories
ImmPRESS-AP Anti-Goat IgG (alkaline phosphatase) Polymer Detection Kit	RRID:AB_2828010	Vector Laboratories
ImmPACT Vector Red Substrate Kit	RRID:AB_2336524	Vector Laboratories
Tween-20	---	VWR
NP40	---	VWR
10% neutral buffered formalin	---	VWR
CDH5-Cre^{ERT2}; Rosa26-tdTomato	---	Yosuke Mukoyama (NHLBI)
C57BL/6J mice	RRID:IMSR_JAX:000664	Jackson Laboratories
AXT Cells	---	Takatsune Shimizu (Hoshi University)
RNA/DNA/Protein Purification Plus kit	---	Norgen Biotek
