# Supplementary material for: Integrated multi-omic analysis of pediatric metastatic osteosarcoma reveals endothelial cell plasticity and lineage infidelity
Source: bioRxiv. 2026 Aug 12:2026.08.11.744222. Preprint. [Version 1] doi: 10.64898/2026.08.11.744222 (PMC13640707; doi:10.64898/2026.08.11.744222)
Supplement: Supplementary file 4 [file media-4.pdf]

Packages	RRID
BSgenome_1.76.0	RRID:SCR_024230
BSgenome.Hsapiens.UCSC.hg38_1.4.5	RRID:SCR_027738
cellranger-arc 2.0.0	RRID:SCR_023897
chromvar_1.30.1	RRID:SCR_026570
ComplexHeatmap_2.24.1	RRID:SCR_017270
CytoTRACE2_1.1.0	RRID:SCR_022828
dittoSeq_1.20.0	
dplyr_1.1.4	RRID:SCR_016708
DRAGEN BCL Convert 4.2.7	
EnhancedVolcano_1.29.1	RRID:SCR_018931
EnsDb.Hsapiens.v86_2.99.0	
ensembldb_2.32.0	RRID:SCR_019103
epiAneuFinder	RRID:SCR_026269
GenePattern	RRID:SCR_003201
ggplot2_4.0.1	RRID:SCR_014601
ggraph_2.2.2	RRID:SCR_021239
GraphPad Prism_10.6.1	RRID:SCR_002798
harmony_1.2.4	RRID:SCR_022206
igraph_2.2.1	RRID:SCR_019225
IRanges_2.42.0	RRID:SCR_006420
JASPAR2020_0.99.10	RRID:SCR_003030
leidenAlg_1.1.5	
MACS2_2.2.9.1	RRID:SCR_013291
Morpheus by Broad Institute	RRID:SCR_017386
motifmatchr_1.30.0	RRID:SCR_026739
msigdbr_25.1.1	RRID:SCR_022870
paletteer_1.7.0	
patchwork_1.3.2	RRID:SCR_024826
plyr_1.8.9	RRID:SCR_026985
presto_1.0.	
RColorBrewer_1.1-3	RRID:SCR_016697
RENEE 2.6.7	
RSEM 1.3.3	RRID:SCR_000262
Scanpy 1.8.2	RRID:SCR_018139
scCustomize_3.2.2	RRID:SCR_024675
SCENIC+ 1.0a2	RRID:SCR_026702
SCEVAN_1.0.1	
scrublet_0.2.3	RRID:SCR_018098
scType	RRID:SCR_026634
Seurat_5.3.1	RRID:SCR_016341
SeuratExtend_1.2.5	RRID:SCR_026143
Signac_1.16.0	RRID:SCR_021158
SingleCellExperiment 1.24.0	RRID:SCR_026794
Snakemake 8.5.5	RRID:SCR_003475
SoupX_1.6.2	RRID:SCR_019193
STAR 2.7.6a	RRID:SCR_004463
TFBSTools_1.46.0	RRID:SCR_024260
tibble_3.3.1	RRID:SCR_026493
tidygraph_1.3.1	RRID:SCR_027617
tidyr_1.3.2	RRID:SCR_017102
tidyverse_2.0.0	RRID:SCR_019186
UCell_2.12.0	RRID:SCR_027109
viridis_0.6.5	RRID:SCR_016696
