# Supplementary material for: Integrated multi-omic analysis of pediatric metastatic osteosarcoma reveals endothelial cell plasticity and lineage infidelity
Source: bioRxiv. 2026 Aug 12:2026.08.11.744222. Preprint. [Version 1] doi: 10.64898/2026.08.11.744222 (PMC13640707; doi:10.64898/2026.08.11.744222)
Supplement: Supplementary file 5 [file media-5.pdf]

**Cell Type**	**Gene List**
Osteoclasts	ACP5, CTSK, ITGB3, MMP13, MMP9
Osteosarcoma Cells	RUNX2, ALPL, SP7, SATB2, CDH11, IBSP, COL1A1, SOX9, ACAN, PTH1R, COL27A1, VDR, POSTN, ASPN, VIM
Myeloid Cells	SPI1, LGALS1, COTL1, MS4A6A, SRGN, TYROBP, LYZ, FCER1G, LAPTM5, AIF1
T Cells	CD3D, CD3E, CD3G, CD8A, CD2, TRAC, CD4
Smooth Muscle Cells	DES, SOD3, MYL9, ACTA2, MGP, CALD1, TPM1, TAGLN, IGFBP7, TPM2
B Cells	CD79A, MS4A1, CD79B, BANK1, CD37, CD22
Fibroblasts	BGN, DCN, MGP, SPARC, CALD1, LUM, COL6A1, IGFBP7, COL1A2, C1S, ACTA2, FAP, VIM
Erythroctye Precursors	HBG2, HBA1, HBA2, HBG1, ALAS2, SPTA1
Lung Epithelial Cells	SFTPB, SFTPC, SFTA3, WIF1, SFTPA1, EPCAM, CDH1
Blood EC	PTRF, CLDN5, AQP1, PECAM1, NPDC1, VWF, RAMP3, RAMP2, SPARCL1, CLEC14A, CDH5, ACKR1
Lymphatic EC	PPFIBP1, GNG11, RAMP2, CCL21, MMRN1, IGFBP7, SDPR, TM4SF1, CLDN5, ECSCR, FLT4, PLVAP
