# Supplementary material for: Integrated multi-omic analysis of pediatric metastatic osteosarcoma reveals endothelial cell plasticity and lineage infidelity
Source: bioRxiv. 2026 Aug 12:2026.08.11.744222. Preprint. [Version 1] doi: 10.64898/2026.08.11.744222 (PMC13640707; doi:10.64898/2026.08.11.744222)
Supplement: Supplementary file 6 [file media-6.pdf]

tdTomato PCR:

PCR Cycling Conditions			
Step 1:	94C for 2 min		
Step 2:	94C for 20 sec	10 cycles	
Step 3:	65C for 15 sec (-0.5C per cycle)		annealing
Step 4:	68C for 10 sec		extention
Step 5:	94C for 15 sec	28 cycles	
Step 6:	60C for 15 sec		annealing
Step 7:	68C for 10 sec		extention
Step 8:	68C for 3 min		
Step 9:	4C hold		

**Primers**	**Sequence**	**Amplicon Size**
IMR9020	AAG GGA GCT GCA GTG GAG TA	297bp (WT)
IMR9021	CCG AAA ATC TGT GGG AAG TC	
IMR9103	GGC ATT AAA GCA GCG TAT CC	196 bp (Mutant)
IMR9105	CTG TTC CTG TAC GGC ATG G	

CreER PCR:

PCR Cycling Conditions			
Step 1:	94C for 2 min		
Step 2:	94C for 15 sec	30 cycles	
Step 3:	60C for 15 sec		annealing
Step 4:	68C for 15 sec		extention
Step 5:	68C for 3 min		
Step 6:	4C hold		

**Primers**	**Sequence**	**Amplicon Size**
Cre FWD	GCGGTCTGGCAGTAAAAACTATC	100bp
Cre RVS	GTGAAACAGCATTGCTGTCACTT	
